# Lung Cancer Therapy Targeting Histone Methylation: Opportunities and Challenges

**DOI:** 10.1016/j.csbj.2018.06.001

**Published:** 2018-06-20

**Authors:** Yuchen Chen, Xinran Liu, Yangkai Li, Chuntao Quan, Ling Zheng, Kun Huang

**Affiliations:** a Tongji School of Pharmacy, Tongji Medical College, Huazhong University of Science & Technology, Wuhan 430030, China; b Tongji Hospital, Tongji Medical College, Huazhong University of Science & Technology, Wuhan 430030, China; c College of Life Sciences, Wuhan University, Wuhan 430072, China

**Keywords:** Histone methylation, Histone demethylation, Lung cancer, Histone methyltransferase, Histone demethylase, Inhibitors, ALK, anaplastic lymphoma kinase, DUSP3, dual-specificity phosphatase 3, Elk1, ETS-domain containing protein, EMT, epithelial-to-mesenchymal transition, HDAC, histone deacetylase, IHC, immunohistochemistry, KDMs, lysine demethylases, KLF2, Kruppel-like factor 2, KMTs, lysine methyltransferases, LSDs, lysine specific demethylases, MEP50, methylosome protein 50, NSCLC, non-small cell lung cancer, PAD4, peptidylarginine deiminase 4, PCNA, proliferating cell nuclear antigen, PDX, patient-derived xenografts, PRC2, polycomb repressive complex 2, PRMTs, protein arginine methyltrasferases, PTMs, posttranslational modifications, SAH, S-adenosyl-L-homocysteine, SAM, S-adenosyl-L-methionine, SCLC, small cell lung cancer, TIMP3, tissue inhibitor of metalloproteinase 3

## Abstract

Lung cancer is one of the most common malignancies. In spite of the progress made in past decades, further studies to improve current therapy for lung cancer are required. Dynamically controlled by methyltransferases and demethylases, methylation of lysine and arginine residues on histone proteins regulates chromatin organization and thereby gene transcription. Aberrant alterations of histone methylation have been demonstrated to be associated with the progress of multiple cancers including lung cancer. Inhibitors of methyltransferases and demethylases have exhibited anti-tumor activities in lung cancer, and multiple lead candidates are under clinical trials. Here, we summarize how histone methylation functions in lung cancer, highlighting most recent progresses in small molecular inhibitors for lung cancer treatment.

## 1 Introduction

Lung cancer is one of the most prevalent malignancies and the leading cause of cancer-related death in the US and in China [1, 2]. Among patients with lung cancer, non-small cell lung cancer (NSCLC) accounts for about 85% and small cell lung cancer (SCLC) accounts for the remaining 15% [3]. According to the pathological phenotypes, NSCLC includes adenocarcinoma, squamous cell carcinoma, and large cell lung carcinoma [4]. The past decades have witnessed the clinical introduction of several small molecular inhibitors, which significantly improved overall prognosis of lung cancer [[5], [6], [7]]. However, many patients still suffer from poor response to drug therapy due to individually differences in genetic, epigenetic, phenotypic or psychosocial features [8, 9]. Therefore, precision medicine aiming to provide personalized targeted treatment becomes an attractive strategy to improve the drug efficacy against lung cancer [[10], [11], [12]]. Epigenetic differences among patients have been considered as a critical factor in the development of precision medicine [13], and in various malignancies including lung cancer, epigenetic dysregulation has been identified to play a crucial role in the tumorigenicity and heterogeneity [[14], [15], [16], [17]].

Epigenetic dysregulation is usually resulted from aberrant changes in DNA and histone modifications. In eukaryotic cell, genomic DNA is wrapped around a protein octamer which contains four core histones (H2A, H2B, H3, H4), forming the structure of the nucleosome [18]. Each of the histone proteins possesses a tail, which is a classic location where various posttranslational modifications (PTMs) function [19, 20]. Through changing the charge density between DNA and histones, DNA methylation and histone PTMs (acetylation, methylation, and phosphorylation) can regulate the loosening of the nucleosome, affecting the access of transcription factors and RNA polymerase to their target genes [[21], [22], [23], [24]]. Currently, DNA methylation has been widely accepted as important biomarkers in the clinical management of lung cancer, since DNA methylation-based biomarkers provide useful information in distinct clinical questions about early diagnosis, staging, prognosis and therapy-response prediction [25]. However, questions about whether other epigenetic modifications can be explored as lung cancer therapeutic targets never stopped during the last decade. Histone acetylation has been demonstrated to play a vital role in lung cancer development by activating gene transcription [16]. Although some histone deacetylase (HDAC) inhibitors, such as Vorinostat and Panobinostat, have gained optimistic results in pre-clinical and clinical trials on NSCLC, further studies of HDAC inhibitors in lung cancer are necessary for evaluating their anti-tumor effect [16, 23]. Histone methylation, one of the most well studied patterns among histone modifications, can either promote or inhibit transcription at different gene loci, thus plays a rather complex role in lung cancer [21]. It is believed that the methylation of lysine (K) and arginine (R) residues on histone tails largely determines the chromatin configurations and, hence, biological outcomes [19]. Like other histone modifications, histone methylation is a dynamic process regulated by a series of ‘eraser’ and ‘writer’ enzymes. Methylation ‘erasers’ and ‘writers’ respectively remove and add specific methyl marks crucial for gene expression, genomic stability and cell fate [19]. Methyltransferase ‘writers’ and the corresponding demethylase ‘erasers’ for histone lysine residue are termed as histone lysine methyltransferases/demethylases (KMTs/KDMs for short respectively). For histone arginine residues, the ‘writers’ and ‘erasers’ are histone arginine methyltransferases and histone arginine demethylases respectively.

Some of these histone methylation modifiers have been identified in cancers with altered activities, suggesting their oncogenic or tumor-suppressor roles [19, 26]. Aberrations of histone methylation modifiers have been closely intertwined with lung cancers as well [14, 27]. Moreover, along with the deeper understanding of the patterns and functions of histone methylation in lung cancer, several inhibitors targeting histone methylation modifiers have entered clinical trials [22]. It may be a right time to review and rethink the potential of histone methylation for developing lung cancer therapy, however, there lacks a systematic review about this issue. Here, we discussed the functions and related structural foundations of histone methylation modifiers in lung cancer, and highlighted the most recent progresses in lung cancer therapy targeting histone methylation.

## 2 KMTs and their Roles in Lung Cancer

**Fig. 1:**
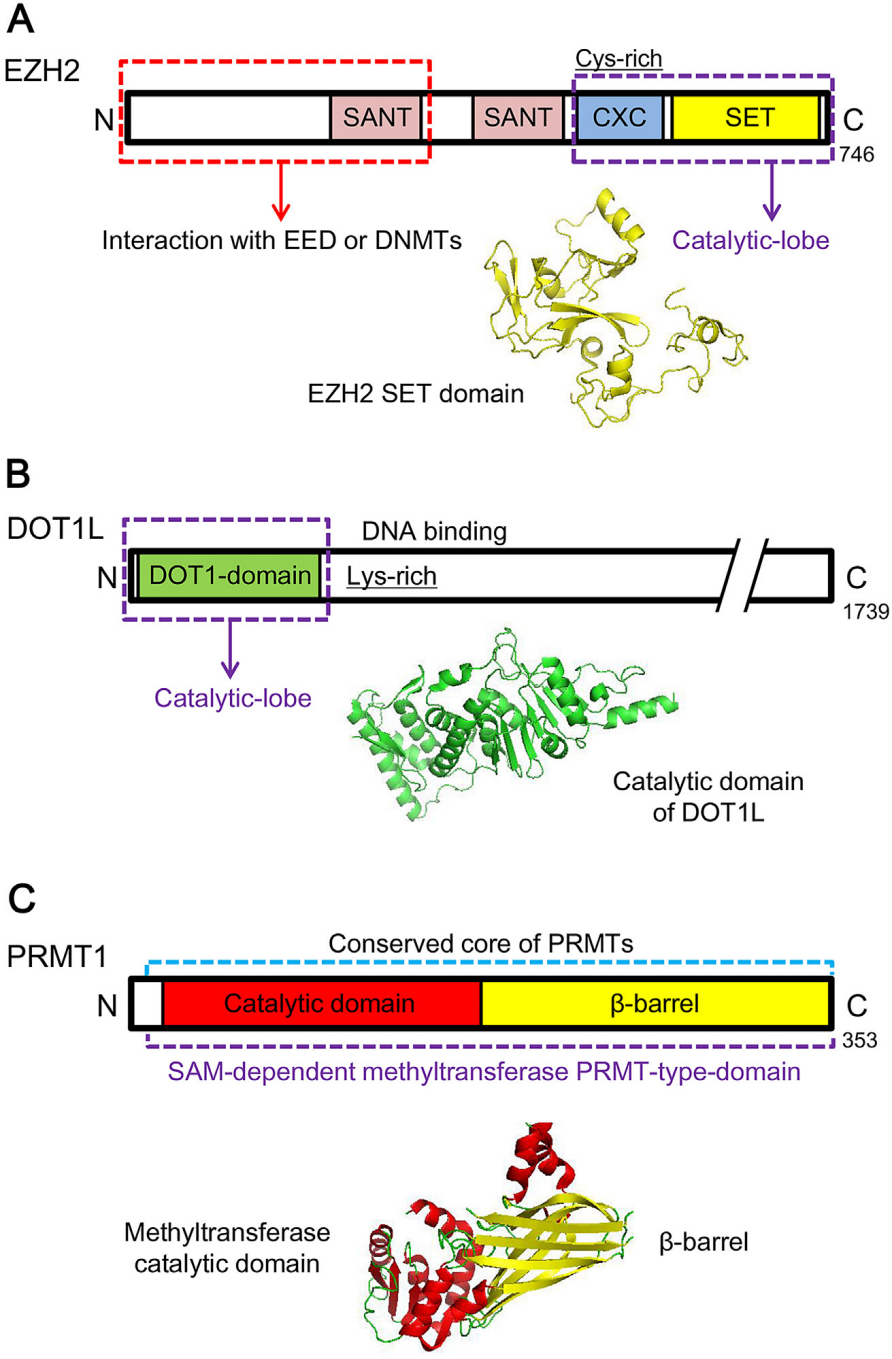
Schematic representations and structures of representative histone methylation ‘writers’. (A) Schematic representations and structure of EZH2. EZH2 belongs to SET-containing KMTs and catalyzes methylation at lysine residues *via* SET domain (PDB ID 4MI5). (B) Schematic representations and structure of DOT1L, the only non-SET-containing KMT enzyme (PDB ID 1NW3). (C) Schematic representations and structure of PRMT1 (PDB ID 1OR8). SANT (Swi3, Ada2, N-Cor, and TFIIIB), a domain that allows chromatin remodeling proteins to interact with histones. CXC, a cys-rich region preceding the SET domain. DNMTs, DNA methyltransferases.

KMTs can remove methyl groups on lysine residues of histones or non-histone substrates [28, 29]. Based on the similarity of structural organization and catalytic domain, KMTs are divided into two categories, SET domain-containing KMTs and the only non-SET-domain-containing KMT DOT1L (Fig. 1A and B) [30, 31]. The first histone KMTs identified in human is the H3K9 methyltransferase SUV39H1, a mammalian homologue of *Drosophila* Su(var 3–9) [32]. Since then, more histone KMTs have been discovered, which target H3K4 [33], H3K9 [26, 34], H3K27 [35, 36], H3K36 [37], H3K79 [38, 39] or H4K20 [40]. In addition to their essential roles in physiologic activities, such methyltransferases are found to closely associate with variant cancers. Here, the structures and functions of representative histone methyltransferases and their therapeutic potentials for lung cancer are summarized (Table 1).

**Table 1:** Histone methyltransferases with reported functions in lung cancer.

Name	Target	Links to lung cancer
MLL2	H3K4	Loss of expression and deleterious mutations in NSCLC [63]
G9a	H3K9	Overexpressed in lung cancers [185]
EZH2	H3K27	Overexpressed in lung cancers [50, 51]
SMYD2	H3K36	Contributed to NSCLC cell growth [73]
SETD2	H3K36	Deleterious mutations in primary NSCLC [74]
WHSC1L1	H3K36	Over expressed in lung cancer [186]
DOT1L	H3K79	Contributed to NSCLC cell growth [84]
SETD8/PRSET7	H4K20	Overexpressed in lung cancer [76]
SUV4-20H1/2	H4K20	H4K20me3 decreased during tumor progression [187]
PRMTs	Arginine on H3 and H4	Contributed to NSCLC cell growth and overexpressed in TKI-resistant NSCLC [135]

### 2.1 SET Domain-Containing KMTs

The SET domain comprises approximately 130 residues, and is regarded as the evolutionarily conserved catalytic motif of KMTs (Fig. 1A). It was originally identified from three *Drosophila* proteins, *i.e.* Suppressor of variegation 3–9 (Su(var) 3–9), Enhancer of zeste (E (z)) and Trithorax (Trx), which involve in epigenetic process [41]. The SET domains of most of histone KMTs bind to histones as well as methyl donors (S-adenosyl-L-methionine, also known as AdoMet or SAM) and reaction products (S-adenosyl-L-homocysteine, also known as AdoHcy or SAH) [42]. Most SET-containing histone KMTs function SAM-dependently or SAH-dependently. A knot-like structure within the SET domain contributes to form the methyltransferase active site where lysine methylation tends to occur [43]. Documented aberrant SET-domain containing KMTs in lung cancer are reviewed below.

#### 2.1.1 EZH2

EZH2, the human homologue of *Drosophila* En (zeste), is the key catalytic component of the Polycomb repressive complex 2 (PRC2). With the help of the cofactors SUZ12 and EED in a SAM-dependent manner, EZH2 plays a pivotal role of transferring one, two and three methylation marks to H3K27 (H3K27me1, me2, me3) (Fig. 3A) [[44], [45], [46]].

High levels of EZH2 and correlated H3K27me3 are closely related to the poor clinical outcome of cancers, including lower overall survival and disease-free survival [[47], [48], [49], [50], [51]]. Additionally, advanced NSCLC patients with positive EZH2 expression compared with those with negative EZH2, showed resistance to platinum-based chemotherapy [52].

Over-expressed EZH2 promotes lung cancer progression in multiple ways involving proliferation, apoptosis inhibition, migration and metastasis. Studies demonstrated a mutual regulation between EZH2 and the vascular endothelial growth factor-A (VEGF-A) signaling pathway and AKT phosphorylation, which closely linked to enhanced cell proliferation, migration and metastasis [53, 54]. Increased EZH2 expression was also found to be closely associated with either E2F amplification or loss of RB1, which induced disruption of the E2F/Rb pathway, in 96% SCLC samples [55]. Aberrant methylation of PRC2 target genes contribute to generate a ‘stem-cell like’ hypermethylation signature in SCLC, leading to highly aggressive tumor phenotype such as rapid cell growth [55]. Moreover, elevated EZH2 expression promoted SCLC progression, by suppressing apoptosis through epigenetically silencing TGF-β type II receptor (TβRII) [56]. Coordinately, silencing EZH2 inhibited lung cancer cell growth by cell cycle disruption and triggering cell death [[57], [58], [59]]. The oncogenic role of EZH2 in lung cancer was now clearly demonstrated by all these works, and several EZH2 inhibitors under clinical trials exhibited potential to be applied as novel targeted therapy or as an aid to current drug therapy for lung cancer (more detailed discussion in 5.1, 5.2) [60, 61]. The results of clinical trials and further mechanistic investigations would be essential for the potential application of EZH2 inhibitors in lung cancer treatment.

#### 2.1.2 MLL2

The MLL (KMT2) methyltransferases family members, which specifically methylate H3K4, are implicated in various cancers either by dysregulation or loss of function [62]. A whole-exome sequencing identified MLL2 as one of the most frequent mutated genes in Chinese NSCLC patients [63]. Loss of MLL2 expression was commonly observed in NSCLC, and deleterious mutations of MLL2 were found in 11.4% NSCLC patients [63]. MLL2 mutations in human SCLC cell lines was associated with reduced MLL2 protein levels and reduced monomethylation of H3K4, and frequent inactivated mutations of MLL2 were also identified in some of primary SCLC clinical samples and SCLC cell lines [64]. However, a recent study found that MLL2 mutation was associated with reduced survival in NSCLC but not in SCLC, indicating that MLL2 may act differently in different lung cancer subtypes [65]. Since MLL2 mutations usually resulted in genome instability [66], mutant MLL2 might drive the initiation of lung cancer, yet this assumption needed to be verified with more evidences.

#### 2.1.3 G9a

G9a is a KMT responsible for the mono- and di-methylation of H3K9 (H3K9me1, me2) [67]. Highly expressed G9a was observed in aggressive lung cancer cells and the progression of mouse lung cancer induced by urethane [68, 69]. RNAi-mediated G9a silencing reduced cell migration and invasion *in vitro* and *in vivo* [68]. Mechanistic investigations revealed that G9a knockdown suppressed the cell adhesion molecule Ep-CAM by reducing the levels of H3K9me2 and disrupting the recruitment of transcriptional cofactors at the Ep-CAM promoter [68]. Similar mechanism was found between G9a and EMT-related proteins, according to another study which demonstrated the metastasis-promoting role of G9a in lung cancer cells [70]. G9a also silenced caspase 1 (CASP1) by increasing the levels of H3K9me2 around CASP1 promoter, thereby promoted NSCLC cell growth and invasion [71]. Moreover, over-expression of G9A or low expression of CASP1 was strongly correlated with poor overall survival in lung cancer [71]. Apart from the oncogenic role of G9a in lung cancer discussed above, G9a inhibition was reported to potentiate the anti-tumor activity of DNA double-strand break (DSB) inducing agents [72]. Further studies may be necessary to investigate whether G9a plays a role in resistance to chemotherapy in lung cancer.

#### 2.1.4 SMYD2 and SETD2

SET and MYND domain-containing 2 (SMYD2) is one of the H3K36-specific methyltransferases, which methylates lysine residues in anaplastic lymphoma kinase (ALK) and contributes to oncogenic ALK activation. Combination treatment of a SMYD2 inhibitor LLY-507 and an ALK inhibitor crizotinib exhibited significantly enhanced suppression on NSCLC cell growth compared with mono-treatment of either agent [73].

Interestingly, unlike SMYD2, another H3K36-specific methyltransferase SETD2 act as a tumor suppressor in lung cancer. Deleterious mutations SETD2 were detected in primary NSCLC tumors [74]. Loss of SETD2 and subsequent decrease of H3K36me3 led to significant tumor-promoting consequences, accelerating both early- and late-stage lung adenocarcinoma tumors in mice [75]. The results indicate that SETD2 may be further explored as a diagnostic or prognostic marker for NSCLC. Moreover, the examples of different roles of H3K36-specific KMTs in lung cancer implicate a complex and precisely orchestrated regulation network for different target genes mediated by the same H3K36 methylation.

#### 2.1.5 SETD8

SETD8, known as KMT5A or SET8, specifically targets H4K20 for methylation and has been implicated in multiple cancer processes [76]. Previous studies revealed that SETD8 was related with proper cell cycle progression, DNA damage response, and transcriptional regulation. Elevated expression of SETD8 stimulated S-phase progression *via* methylating a non-histone protein proliferating cell nuclear antigen (PCNA), thus promoted proliferation of lung cancer cells [76]. Additionally, SETD8 was directly inhibited by a tumor suppressor miR-382 in NSCLC cells, which led to inhibition in NSCLC cell tumorigenesis and metastasis [77]; whereas restoration of SETD8 can enhance NSCLC cell proliferation, migration and invasion *in vitro* [77]. SETD8 was also reported to reprogram cancer cell metabolism *via* hypoxia-inducible factor-1α (HIF-1α) mediated process by stabilizing HIF-1α protein through post-transcriptional regulation [78]. Meanwhile, SETD8 was also capable of monomethylating the tumor suppressor p53 on lysine 382, which can attenuate the pro-apoptotic and growth arrest functions of p53 [79]. Taken together, SETD8 seems to function in lung tumorigenesis and metastasis beyond a mere histone methyltransferase role.

### 2.2 DOT1L, a Non-SET-Domain-Containing KMT

The catalytic activity of SET domain is not the only determining factor of KMTs function [22, 80]. DOT1L is the only known H3K79 methyltransferase, and shares structural similarities with type I protein arginine methyltrasferases (PRMTs) [81, 82]. Through a spatial arrangement at its N terminal, DOT1L catalyzes SAM-dependent methylation on nucleosomal substrates (Fig. 1B and 3B) [81]. DOT1L and H3K79me3 were identified as promising targets for the treatment of acute myelocytic leukemia (AML), but played a rather unclear role in lung cancer [83].

H3K79 methylation was up-regulated in lung cancer cell lines and clinical tumor tissues. DOT1L knockdown reduced H3K79 methylation and led to disturbed cell proliferation; additionally, chromosomal missegregation occurred in DOT1L-deficient lung cancer cells, resulting in cell cycle arrest at G1 phase and subsequent senescence [84]. Interestingly, during the process of TGF-β1-induced epithelial-to-mesenchymal transition (EMT) in lung cancer, H3K79me3 was decreased without association with DOT1L expression; and DOT1L inhibitors, EPZ5676 and SGC0946, were not effective on EMT-related genes [85]. These seemingly contradictory results indicate a complex mechanism for H3K79 methylation in lung cancer, more studies are required to establish a link between different roles of DOT1L and H3K79 methylation.

### 2.3 Other KMTs

Reports about the role of other KMTs in lung cancer are relatively rare. Studies in cancer cell lines might provide a glimpse of their roles, revealing directions to further investigations. For examples, H4K20-specific KMTs SUV4-20H1/2 played an important role in maintaining genomic stability through methylating H4K20 to H4K20me2 and H4K20me3 [86]; WHSC1L1, a KMT for H3K36, enhanced activation of ERK pathway by mono-methylating lysine 721 of the tyrosine kinase domain of epidermal growth factor receptor (EGFR) [87]. These discoveries suggested that there are remain multiple unknowns about KMTs in lung cancer. More KMTs, like above-mentioned, with unrevealed yet study-worthy functions in lung cancer will be identified in future research.

## 3 KDMs and their Roles in Lung Cancer

Methylation on protein lysine or arginine residues was not regarded as a reversible PTM until the discovery of the first histone demethylase in 2004 [88, 89]. Based on the oxidative mechanism of the demethylation reaction and the structure of the catalytic domains, KDMs can be categorized into lysine specific demethylases (LSDs, or KDM1 subfamily) and Jumonji (JmjC)-domain-containing demethylases (JmjC KDMs, or KDM2–7 subfamilies). So far, >20 KDMs have been discovered and characterized, and many of them have been reported to be dysregulated in multiple diseases [90, 91]. Here, we summarize representative aberrant KDMs in lung cancer (Table 2).

**Table 2:** Histone demethylases with reported functions in lung cancer.

Name	Target	Links to lung cancer
KDM1A,LSD1	H3K4me2/me1, H3K9me2/me1	Overexpressed in lung cancer [97, 98]
KDM2A	H3K36me2/me1	Overexpressed in NSCLC [105]
KDM3A	H3K9me2/me1	Overexpressed in NSCLC [108]
KDM4A	H3K9me3/me2,H3K36me3/me2	Overexpressed in lung cancer [115]
KDM4C	H3K9me3/me2,H3K36me3/me2	Overexpressed in lung sarcomatoid carcinoma [188]
KDM4D	H3K9me3/me2/me1,H3K36me3/me2	Overexpressed in lung cancer [115]
KDM5A	H3K4me3/me2	Overexpressed in lung cancer [117]
KDM6A	H3K27me3/me2/me1	Loss led to lung tumorigenesis [123]
JMJD6	Arginine on H3 and H4	Overexpressed in lung cancer [137]
PAD4	Arginine on H3 and H4	Overexpression led to gefitinib resistance in NSCLC [143]

### 3.1 Non-JmjC-Domain-Containing KDMs

**Fig. 2:**
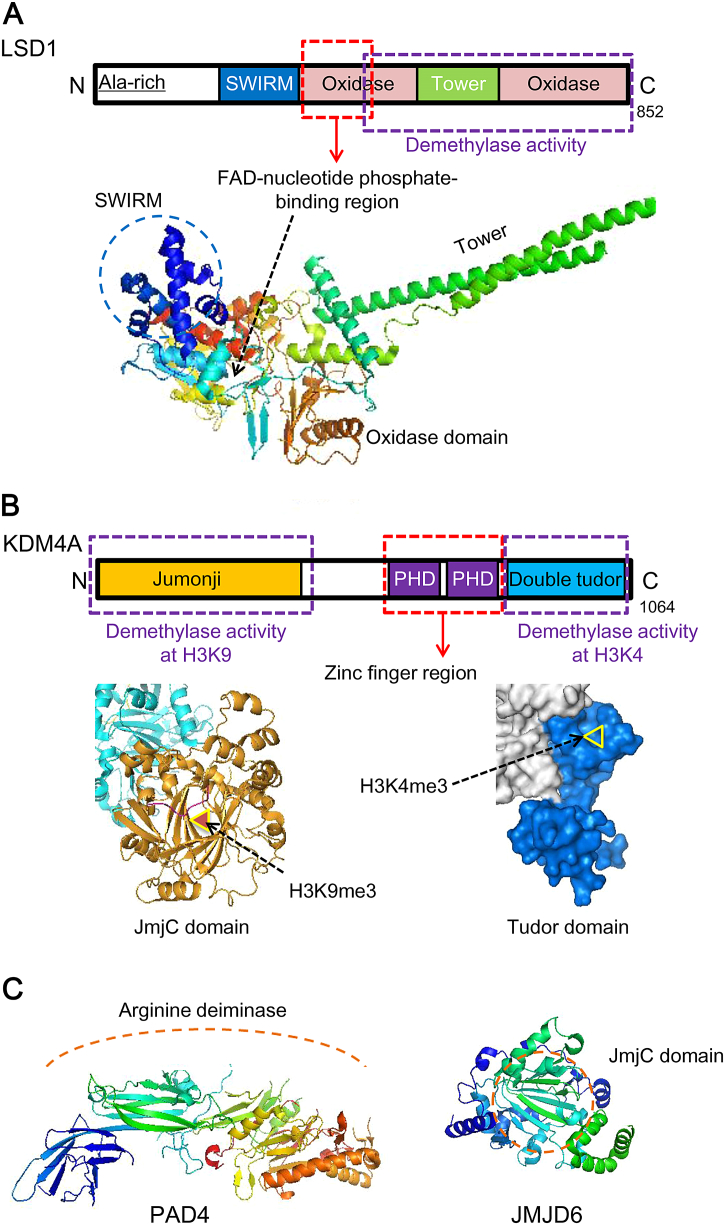
Schematic representations and structures of representative histone methylation ‘erasers’. (A) Schematic representations and structure of LSD1, which functions without JmjC domain (PDB ID 2DW4). (B) Schematic representations and structure of KDM4A, a classic JmjC family demethylase. KDM4A can remove methyl groups from either H3K9 or H3K4 *via* reactions on different sites (JmjC domain and H3K9me3, PDB ID 2OX0. Tudor domain and H3K4me3, PDB ID 2GFA). (C) Secondary structures of histone arginine ‘demethylases’ PAD4 (PDB ID 3APN) and JMJD6 (PDB ID 3LD8). The schematic arrangement of PAD4 is relatively insufficient, and JMJD6 shares similar schematic structure with other JmjC family demethylases. SWIRM (Swi3, Rsc, and Moira) domain, a proposed anchor site for histone molecules. PHD, hydrophobic cage of residues that bind methylated peptides.

**Fig. 3:**
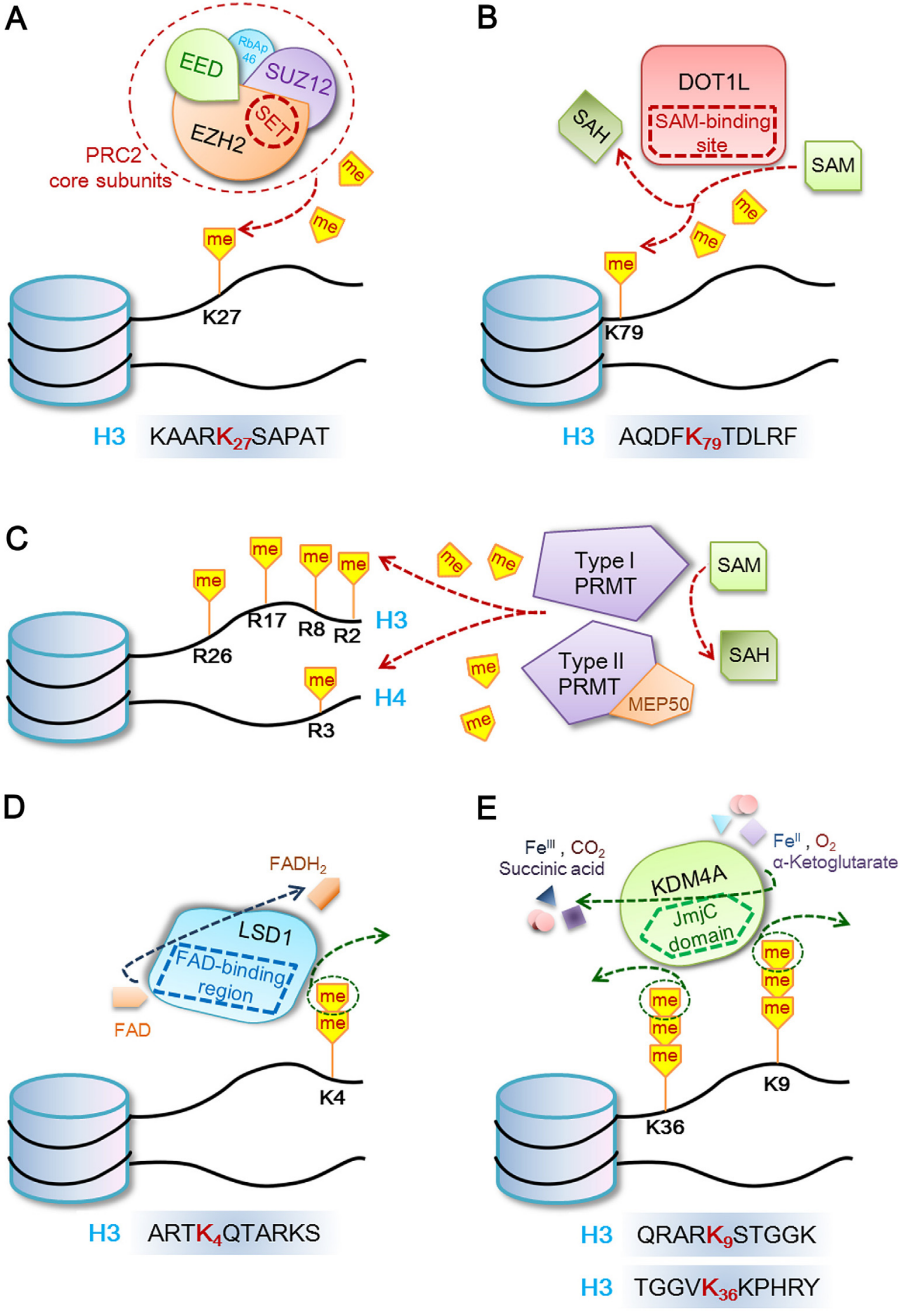
Cartoons of representative histone methylation and demethylation reaction catalyzed by different enzymes. (A) SET-containing EZH2 as a PRC2 core component methylates H3K27 *via* SET domain. (B) Non-SET-domain-containing DOT1L methylates H3K79 SAM-dependently. (C) SAM-dependent arginine methylation on histone H3 and H4. (D) FAD-dependent H3K4me2/me1 demethylation by non-JmjC KDM LSD1. (E) JmjC-containing KDM4A demethylates H3K36me3/me2 and H3K9me3/me2 *via* JmjC-domain-mediated reaction involving αKG and Fe(II).

The LSD family members demethylate lysine residues with a cofactor flavin adenine dinucleotide (FAD) (Fig. 2A) [92, 93]. Interestingly, because the forming of an imine intermediate requires protonated amine during the demethylation process, the LSD family members are only capable of demethylating mono- and dimethyl lysine residues (me1, me2), but not trimethylated (me3) lysine residues (Fig. 3D) [89].

LSD1 (or KDM1A), the first reported and most studied KDM demethylase, belongs to the non-JmjC-domain-containing LSD family [88]. LSD1 usually functions on H3K4 as a key component of the CoREST complex, yet it may change target to H3K9 with the presence of the androgen receptor, thus acting either as a transcriptional corepresssor or a coactivator [94, 95].

As a H3K4 and H3K9 KDM enzyme, LSD1 demonstrated aberrant overexpression and acted as a classic oncogene in various cancers including lung cancer [96]. Overexpressed LSD1 was closely correlated with shorter overall survival of NSCLC patients [97]. Consistently, LSD1 silencing resulted in significant suppression of proliferation of lung cancer cell lines [96]; moreover, SCLC was sensitive to a LSD1 inhibitor GSK-2879552 [98]. LSD1 was recruited to Kruppel-like factor 2 (KLF2) or E-cadherin promoters *via* binding with several lncRNAs in NSCLC cells, resulting in promoted tumor proliferation and EMTs [99, 100]. Expression of tissue inhibitor of metalloproteinase 3 (TIMP3) was repressed by LSD1-mediated H3K4me2 demethylation at TIMP3 promoter, which consequently enhanced MMP2 expression as well as JNK phosphorylation, and eventually promoted the metastasis of NSCLC cells [101]. A LSD1/ integrin β3 axis was also reported to attribute to tumor progression and invasiveness in lung adenocarcinoma [102]. Due to its critical roles in promoting lung cancer as well as various developed specific inhibitors, LSD1 is regarded as a highly promising target for treating lung cancer.

### 3.2 JmjC KDMs

Unlike LSD family, JmjC KDMs have been proven to remove all three methylation states from lysine residues [103]. The JmjC KDMs catalyze demethylation utilizing Fe(II) as a cofactor, and 2-oxoglutarate (2-OG) and α-ketoglutarate (αKG) as co-substrates (Fig. 3E) [90, 92]. The JmjC domain, folding into eight β-sheets, provides an active pocket for αKG and Fe(II) binding [93]. Dysregulation of JmjC KDMs has been observed in different cancers. Representative JmjC KDMs associated with lung cancer are discussed below.

#### 3.2.1 KDM2A

KDM2 catalyzes demethylation on H3K36, which is associated with gene activation [104]. KDM2A, but not its homologue KDM2B, was reported to be associated with lung cancer. KDM2A was found highly dysregulated in 54 NSCLC cell lines according to Affymetrix microarray gene expression data, and its mRNA and protein levels are significantly higher in primary NSCLC tumor samples than in adjacent normal lung tissues [105]. KDM2A-catalyzed H3K36me2 demethylation occurred gene-specifically at the promoter region of cancer-related genes including dual-specificity phosphatase 3 (DUSP3), which in turn antagonized DUSP3-mediated ERK1/2 dephosphorylation and consequently promoted lung tumorigenesis [105]. KDM2A also transcriptionally repressed the histone deacetylase 3 (HDAC3) by demethylating H3K36me2 at the HDAC3 promoter, thereby up-regulated HDAC3 target genes including the cell invasion-associated NANOS1 and the cell cycle-related CDK6 in KDM2A-overexpressing NSCLC cells [106]. However, the lack of ideal inhibitors to KDM2A limits the potential amplification of KDM2A-based therapy. Recent discovery of highly selective inhibitor of KDM2A might provide opportunities to develop KDM2A targeted therapy for lung cancer [107].

#### 3.2.2 KDM3A

The expression level of KDM3A, a H3K9-specific KDM demethylase, was found upregulated in more than half of the NSCLC cases [108]. KDM3A activated Homeobox A1 (HOXA1) transcription by removing methyl groups from H3K9me2 at HOXA1 promoter, stimulated the activation of HOXA1 downstream target gene CCND1, an essential factor of the cell cycle progression, thus positively regulated G1/S transition in A549 cell line [108]. Additionally, KDM3A knockdown decreased the expression of tumor-promoting EZH2 and increased the anti-tumor miRNA let-7c expression, thus inhibited tumorigenesis in NSCLC cell lines and xenograft model [109].Recently, KDM3A was also found to facilitate the immune evasion of A549 cells by promoting Foxp3 transcription [110], which provides another evidence that KDM3A acts as an oncogene in lung cancer. Interestingly, KDM3A showed an anti-apoptotic function by erasing monomethylaion from p53K372, thus disturbing the stability of chromatin-bound p53 in cancer and promoted drug resistance [111], which brings new insight to understanding the role of KDM3A in lung cancer.

#### 3.2.3 KDM4A and KDM4D

KDM4 subfamily can demethylate H3 at K4, K9 and K36 residues. KDM4B and KDM4C are structurally and catalytically similar to KDM4A, which is well studied, whereas KDM4D is unique for its lack of PHD and Tudor domains [112]. The JmjC domain in KDM4A is responsible for demethylating H3K9me3/me2 and H3K36me3/me2, and the substrate of Tudor domain is H3K4me3 (Fig. 2B) [113]. Aberration of KDM4 subfamily members were discovered in various cancers including breast cancer and prostate cancer, yet reports on the link between KDM4 subfamily members and lung cancer are relatively rare [22, 114]. Soini et al. found that both KDM4A and KDM4D appeared to significantly relate to the metastasis of lung cancer. They observed more nuclear and cytoplasmic KDM4A expression in the tumors with lymph node metastasis than in tumors with no metastasis, and the same tendency also occurred in nuclear expression of KDM4D. They also discovered an association between cytoplasmic KDM4A expression and poor prognosis both in survival and recurrence free interval [115]. To further understand these observations, additional studies are required to establish a mechanistic link between KDM4A and its oncogenic role in lung cancer.

#### 3.2.4 KDM5A

KDM5A, a H3K4-spesific KDM demethylase, was found to be implicated in developing drug resistance in a study on the epigenetic basis of cancer drug resistance [116], and it was enriched in lung cancer tissues as well as drug-resistant cells [117]. KDM5A bound directly to the promoters of integrin-β1, which was reported to mediate cell-matrix interaction [118, 119], thereby promoted cell migration and invasion. Meanwhile, KDM5A bound to the promoters of cell-cycle-related genes cyclin D1 and p27, promoting cell proliferation directly by repressing p27 and activating cyclin D1 and indirectly up-regulating cyclin E1 expression. Additionally, a selective inhibitor of KDM5A, YUKA1, suppressed cell proliferation and prevented drug resistance in various cancer cell lines including A549 [120], implicating its application potential in lung cancer treatment.

#### 3.2.5 KDM6A

The link between lung cancer and KDM6A, a H3K27-specific demethylase which usually acts antagonistically to EZH2, was previously studied in NSCLC cells with controversial outcomes: KDM6A epigenetically antagonized TGF-β-induced EMT process [121]; whereas its inhibitor GSKJ4 demonstrated anticancer-effect on a set of NSCLC cell lines [122]. However, a recent study unveiled KDM6A as an important tumor suppressor gene in lung cancer with evidence gained from human lung cancer specimens and transgenic NSCLC mouse models [123]. KDM6A knockout resulted in an increased EZH2 level and an up-regulated H3K27me3 level, and significantly promoted lung tumorigenesis *in vivo* [123].

Interestingly, the KDM6A-knockout lung tumors appeared to be more sensitive to EZH2 inhibitor, indicating that NSCLC patients with KDM6A loss may get more benefit from EZH2 inhibition therapy [123]. Such preferentially sensitivity was also identified in other malignant diseases. For examples, loss of KDM6A amplified PRC2-regulated transcriptional repression of IGFBP3 in urothelial bladder cancer and promoted tumor growth, and sensitized bladder cancer cells and tumors to EZH2 inhibition [124]; moreover, loss of KDM6A led to malignant phenotype in multiple myeloma *via* deactivating the expression of multiple genes including IRG4 and c-MYC, and EZH2 inhibitors performed better anti-tumor effects in KDM6A loss cases by rebalancing H3K27me3 levels at specific genes [125]. This balance between KDM6A and EZH2 may be important in guiding therapeutic strategy in multiple diseases, including lung cancer.

### 3.3 Other KDMs

Although other JmjC KDM subfamilies members are supposed to involve in tumoringenesis, for example KDM6B in T-cell acute lymphoblastic leukemia (T-ALL) [126], reports of their roles in lung cancer are very limited. Additionally, there is no report that relates KDM7 subfamily with lung cancer. Future analysis in clinical samples may help identify their roles in lung cancer.

## 4 Histone Arginine Methylation/Demethylation in Lung Cancer

Histone arginine methylation participates in epigenetic regulation largely by cross-talks with other epigenetic modifications [127]. With the capacity to prevent or enhance the binding of important transcriptional factors, histone arginine methylation is found in both repressed and active chromatin states [128].

### 4.1 Histone Arginine Methylation

Methylation on arginine residues is catalyzed by protein arginine methyltransferases (PRMTs) family, which transfers the methyl group from SAM to the guanidino group of arginine [129]. The catalytic core of PRMT consists of a β-barrel (unique to PRMT), a methyltransferase domain, and a dimerization arm (conserved in type I PRMT) [130]. PRMTs are categorized into two types according to their structural similarity. Type I PRMTs, such as PRMT1–4, 6 and 8, catalyze methylation SAM-dependently with a SAM-binding site like DOT1L (Fig. 1C) [131]. The only identified type II PRMT is PRMT5, which forms a protein complex with methylosome protein 50 (MEP50) to exert its catalytic function (Fig. 3C) [21, 128, 132].

PRMT1, PRMT4 and PRMT6 demonstrated higher expression in lung cancer tissues, while abrogation of each resulted in growth suppression [133, 134]. Moreover, PRM1 played a role in lung cancer metastasis. For instance, silencing PRMT1 decreased a mitogenic factor called Neuromedin B receptor while increased epithelial markers cytokeratins 7 and 8, which consequently resulted in reduced cell proliferation and enhanced tumor differentiation [134]. Additionally, up-regulated PRMT1 repressed E-cadherin activity and promoted EMT in erlotinib-resistant NSCLC cells (erlotinib is a tyrosine kinase inhibitor against NSCLC) [135].

### 4.2 Histone Arginine Demethylation

#### 4.2.1 JMJD6

Arginine methylation is very stable, and whether it can be directly demethylated by enzymes remained unclear until the discovery of a putative histone arginine demethylase, JMJD6 [128, 136]. The JmjC-containing JMJD6 was previously known as a phosphatidylserine receptor (Fig. 2C) [128]. Significantly high expression of JMJD6 in lung adenocarcinoma was found positively correlated with tumor size and pleural invasion, and led to significantly poor clinical outcomes [137]. Moreover, an elevated level of JMJD6 was positively associated with pathological grade, pT status and pN status, indicating its potential to be a clinical diagnostic and prognostic marker for NSCLC [137]. Notably, targeting JMJD6 may provide additional ways to treat lung cancer, since suppressing JMJD6 *via* acetylating its upstream transcriptional factor HOXB9 resulted in a decrease in tumor growth and migration in xenograft models [138].

#### 4.2.2 PAD4

Peptidylarginine deiminase 4 (PAD4) targets arginine sites on histone H3 and H4 [139, 140]. Methylation marks on histone arginine residues occasionally convert into citrullination marks *via* PAD4-mediated hydrolysis, representing another form of ‘demethylation’ (Fig. 2C) [141].

PAD4 exacerbated TNF-α-induced lung inflammation [142]. In addition, PAD4 expression decreased significantly in gefitinib-resistant NSCLC cells (gefitinib, a widely-used tyrosine kinase inhibitor against NSCLC) [143]. Overexpression of PAD4 inhibited EMT activity by suppressing ETS-domain containing protein (Elk1), which was reported to regulate EMT process [144], and thus restrained gefitinib resistance [143].

## 5 Representative Histone Methyltransferase/Demethylase Inhibitors

Inhibitors targeting either histone methyltransferases or demethylases have been widely reported to exert anti-tumor activities for multiple malignancies either in singe-agent therapy or combination therapy. In pre-clinical studies, for examples, a classic G9a inhibitor BIX-01294 induced autophagy-associated cell death and impaired tumor growth in breast cancer [145], oral squamous cell carcinoma [146] and hepatocellular carcinoma [[145], [146], [147]]; GSKJ4, the selective inhibitor of KDM6A and KDM6B, not only effectively suppressed tumor progression in AML [148], breast cancer [149], ovarian cancer [150] and castration-resistant prostate cancer [151], but also enhanced the radiosensitivity of multiple tumor cell lines [152]. Some of the inhibitors have entered clinical trials after their anti-cancer potentials identified in pre-clinical studies. For single-agent therapy, some LSD1 inhibitors such as TCP/ATRA are under phase I/II clinical trials for AML therapy (Trial number: NCT0273102, NCT2267). For combination therapy, the most potent DOT1L inhibitor EPZ5676 showed synergy with daunorubicin and cytarabine, two standard agents in current chemotherapy for AML [153, 154]; an EZH2 inhibitor EPZ6438 (tazemetostat), in combination with prednisolone, entered phase II clinical trials for diffuse large B-cell lymphoma (Trial number: NCT01897571). In general, more and more histone methylation modifier inhibitors have been identified as potential reagents aiding cancer therapy. For lung cancer, to the best of our knowledge, inhibitors targeting EZH2 and LSD1 demonstrated most prominent anti-tumor effects (Table 3).

**Table 3:** Representative inhibitors of EZH2 or LSD1 in lung cancer.

Compound	Structure	Mechanism and potency	Clinical Trial Number	Ref
DZNep		SAH hydrolase inhibitor (K_i_ = 50 pM)	N/A	[155, 160]
GSK2816126 (GSK126)		SAM-competitive EZH2 inhibitor (IC50 = 9.9 nM)	NCT02082977	[165, 172]
EPZ6438 (Tazemetostat)		SAM-competitive EZH2 inhibitor (Ki = 2.5 nM) (IC50 = 11 nM)	NCT01897571 NCT02601950 NCT02601937	[167, 172]
CPI1205		SAM-competitive EZH2 inhibitor	NCT02395601	[168, 172]
GSK-2879552		FAD-dependent irreversible LSD1 inhibitor (K_iapp_ = 1.7 μM)	NCT02034123	[98, 176]
RG6016 (ORY-1001)		FAD-dependent irreversible LSD1 inhibitor (IC50 < 20 nM)	NCT02913443	[177]

N/A: Not available.

### 5.1 EZH2 Inhibitors

The structure of the conserved SET domain in EZH2 predicts two critical binding pockets for inhibitors: the key methyl donor SAM and the H3K27 substrate [155]. So far, almost all the small molecular inhibitors targeting EZH2 are SAM-competitive [156]. Although such EZH2 inhibitors are usually obtained *via* diverse independent screens, most of them share a common structure namely pyridine group [22]. Recently, a series of 4-amino-2,2′,6,6′-tetramethylpiperidine analogues, which inhibited EZH2 in a SAM-competitive manner, were identified yet demonstrated weaker cellular potency comparing to the pyridone-based inhibitors [157, 158]. EZH2 inhibitors are widely applied in pre-clinical and clinical trials of various cancers due to the remarkable link of EZH2 dysregulation to oncogenesis in multiple tissue types [159]. For lung cancer, some EZH2 inhibitors stood out with promising therapeutic potentials.

3-dezaneplanocin-A (DZNep) was the first and best known EZH2 inhibitor identified through drug screening [160]. It is a SAH hydrolase inhibitor, which indirectly inhibits the methylation reaction *via* interfering with SAM and SAH metabolism [155]. Investigations on NSCLC cell lines revealed a significant dose-dependent growth inhibitory effect of DZNep [161]. Further investigation using a whole-body physiologically based pharmacokinetic (PBPK) models predicted that DZNep administration at proper dose could exert anti-tumor effect *in vivo* [162]. However, the lack of specificity, which may lead to unwanted SAM-dependent reactions, along with its short half-life, limited the clinical translation of DZNep [163].

Ever since then, more selective EZH2 inhibitors with better anti-lung cancer effects have been identified. For example, GSK2816126 was obtained through optimization of a previously identified compound GSK-A [164], which was SAM-competitive but noncompetitive *versus* histones [165]. GSK2816126 was highly selective to both WT and mutant EZH2 and showed slow inhibitor-enzyme dissociation [165, 166]. GSK2816126 not only inhibited migration of A549 dose-dependently and showed similar effect to gefitinib at the same doses, it also inhibited angiogenesis *in vitro* and *in vivo* [60].

Other representative optimized EZH2 specific pyridone-based inhibitors include EPZ6438 [167] and CPI1205 [168], which showed selective inhibition for EZH2 with improved oral bioavailability [169]. EPZ6438, CPI1205 and GSK2816126 have already entered clinical trials (Trial number: NCT01897571, NCT02601950, NCT02601937, NCT02395601, and NCT02082977) and achieved satisfying positive results in solid tumors, indicating their potential for further investigation in lung cancer [[170], [171], [172]]. Moreover, pharmacological EZH2 inhibition also sensitized lung tumors to other inhibitors. For examples, either DZNep or GSK126 administration promoted the anti-tumor effect of TopoII inhibitor doxorubicin against BRG1 and EGFR mutant lung cancer, suggesting an opportunity for combination of traditional chemotherapy medicines and EZH2 inhibitors in NSCLC [61].

### 5.2 LSD1 Inhibitors

The catalytic domain of LSD1 contains a FAD-binding site. It is a highly conserved functional region indispensible for LSD1-mediated demethylation process [94]. Despite varied chemical characters of presently known LSD1 inhibitors, all LSD1 inhibitors in clinical trials are FAD-dependent irreversible LSD1 inhibitors [173]. Two most potent LSD1 inhibitors, GSK-2879552 and RG6016 (also known as ORY-1001), share a privileged scaffold called tranylcypromine (TCP), indicating that TCP is critical for further designing of LSD1 inhibitors [173]. Although accumulating reports linked LSD1 aberrations with multiple malignancies, the most optimizing results of LSD1 inhibition therapy were obtained in AML and SCLC [174, 175]. Both GSK-2879552 and RG6016 represented a promising novel epigenetic approach for SCLC therapy.

GSK-2879552 was first discovered and characterized in 2015, which irreversibly inactivated the catalytic activity of LSD1 [98]. GSK-2879552 led to enhanced H3K4 methylation at loci of LSD1 target genes in a dose and time dependent manner, thus increased the activation of genes important for cell-development, leading to significant cytostatic effect in various SCLC cell lines and anti-tumor activity in three SCLC xenograft models [98]. In patient-derived xenografts (PDX) models, stronger methylation signatures were associated with increased sensitivity to GSK-2879552 [98, 176]. Following preclinical validation, GSK-2879552 has stepped into Phase I clinical trials for relapsed/refractory SCLC (Trial number: NCT02034123). Similarly, RG6016 inhibited the proliferation of multiple SCLC cell lines and xenograft growth, and has also entered Phase I clinical trials for SCLC therapy (Trial number: NCT02913443). Interestingly, RG6016 treatment was only effective on cell lines and xenografts with certain similar gene expression pattern which also observed in SCLC patient samples, indicating that RG6016 responsive gene signature may help identifying SCLC patients who may benefit from LSD1-based therapy [177].

## 6 Challenges and Future Directions

Alteration of histone methylation patterns is widely proved to play a vital role in multiple malignancies. Recent progress made in this field has drawn attention to the role of histone methylation in lung cancer. Dysregulation of histone methylation ‘writers’ and ‘erasers’ are closely linked to clinical outcomes in lung cancer patients through a variety of cellular pathways relating proliferation, invasion, EMT *etc.* Some histone methylation modifiers, such as SETD8, KDM2A and KDM6A, were identified as oncogenes or tumor suppressor genes in lung cancer [76, 105, 123]; some demonstrated potential link with drug resistance to lung cancer therapy, for example KDM5A and KDM3A [111, 117]. JMJD6 and KDM4A might serve as biomarkers for lung cancer although further mechanistic investigations were necessary [115, 137]. Lung tumors with KDM6A loss was more sensitive to EZH2 inhibition, indicating lung cancer patients with specific histone methylation features might benefit from specific epigenetic therapy [123]. Despite that some questions, such as how H3K36-specific KMTs function differently in lung cancer, remain unclear, inhibitors targeting histone methylation modifiers have gradually entered clinical trials for lung cancer therapy and show optimizing anti-tumor effect in either mono-treatment or combination treatment [60, 61, 178]. All these progress indicate the potential importance of histone methylation for lung cancer.

Although there is no applications in everyday clinical practice yet, in depth studies on the histone methylation dysregulation of lung cancer not only help better understand the mechanism of tumorigenesis and development of lung cancer, but also lead to discovery of potential molecular targets, biomarkers, and therapeutic candidates for lung cancer. Additionally, lung cancer with certain histone methylation features may provide new fine classifications in lung cancer that eventually leads to precise personal medicine. Moreover, the development of histone methylation modifier inhibitors, such as EZH2 or LSD1 inhibitors, not only provides promising therapeutic choices for lung cancer treatment, but also may benefit patients who are resistant to current targeted therapies like tyrosine kinase (*e.g.* EGFR) inhibition by combining with chemotherapy [61, 173].

Meanwhile, challenges still exist and have revealed directions for future research. First, targeting one histone methyl residue or one certain related enzyme may result in diverse unpredictable effects. This is because the interactions between histones, histone methyltransferases and histone demethylases appear to be influenced by the subtle environment surrounding the residue, and crosstalk between different methylated sites are also involved [179]. For example, H3K9me3 prevented SET7-dependent mono-methylation on H3K4 [180], and H3K4me3 prevented G9a- or other H3K9 KMTs-mediated methylation on H3K9 [179, 181]. Second, the lack of complete information and thorough understanding about histone methylation remains an obstacle to further developing epigenetic strategies for lung cancer. For instance, until now, H3K79-specific KDMs have not been reported yet [182]. Additionally, the regulatory mechanism behind the precise assignment of different methyltransferas/demethylasessjbe is not fully explained [183]. The third challenge is to develop more specific inhibitors with clinical application potential, since most histone methyltransferases or demethylases share similar structures and catalytic domains. Take SET domain for example, it participates in a number of reactions, whereas a majority of KMTs contain this domain [42]. A number of inhibitors targeting SET domain were found lacking specificity, which may lead to unwanted effects [184]. The first two challenges require further mechanistic investigations, which are important to complete the map of histone methylation in lung cancer. To deal with the third challenge, rational drug design is necessary to develop relative inhibitors with high selectivity and satisfactory pharmacokinetics. In addition, more pre-clinical and clinical studies are also required to evaluate the anti-tumor efficacy and side effects of new drugs.

In summary, targeting histone methylation is a promising therapeutic strategy for lung cancer treatment. Ever since the recognition of the significance of epigenetic dysregulation in lung cancer, extensive studies have revealed complex but precisely orchestrated regulations mediated by different histone methyltransferases/demethylases. Along with the inhibitors under study or in trials, these efforts have paved the way for an era of better lung cancer therapy.
